# Structure and function of archaeal histones

**DOI:** 10.1371/journal.pgen.1007582

**Published:** 2018-09-13

**Authors:** Bram Henneman, Clara van Emmerik, Hugo van Ingen, Remus T. Dame

**Affiliations:** 1 Leiden Institute of Chemistry, Leiden University, Leiden, the Netherlands; 2 Centre for Microbial Cell Biology, Leiden University, Leiden, the Netherlands; Washington University in St. Louis, UNITED STATES

## Abstract

The genomes of all organisms throughout the tree of life are compacted and organized in chromatin by association of chromatin proteins. Eukaryotic genomes encode histones, which are assembled on the genome into octamers, yielding nucleosomes. Post-translational modifications of the histones, which occur mostly on their N-terminal tails, define the functional state of chromatin. Like eukaryotes, most archaeal genomes encode histones, which are believed to be involved in the compaction and organization of their genomes. Instead of discrete multimers, in vivo data suggest assembly of “nucleosomes” of variable size, consisting of multiples of dimers, which are able to induce repression of transcription. Based on these data and a model derived from X-ray crystallography, it was recently proposed that archaeal histones assemble on DNA into “endless” hypernucleosomes. In this review, we discuss the amino acid determinants of hypernucleosome formation and highlight differences with the canonical eukaryotic octamer. We identify archaeal histones differing from the consensus, which are expected to be unable to assemble into hypernucleosomes. Finally, we identify atypical archaeal histones with short N- or C-terminal extensions and C-terminal tails similar to the tails of eukaryotic histones, which are subject to post-translational modification. Based on the expected characteristics of these archaeal histones, we discuss possibilities of involvement of histones in archaeal transcription regulation.

## Author summary

Both Archaea and eukaryotes express histones, but whereas the tertiary structure of histones is conserved, the quaternary structure of histone–DNA complexes is very different. In a recent study, the crystal structure of the archaeal hypernucleosome was revealed to be an “endless” core of interacting histones that wraps the DNA around it in a left-handed manner. The ability to form a hypernucleosome is likely determined by dimer–dimer interactions as well as stacking interactions between individual layers of the hypernucleosome. We analyzed a wide variety of archaeal histones and found that most but not all histones possess residues able to facilitate hypernucleosome formation. Among these are histones with truncated termini or extended histone tails. Based on our analysis, we propose several possibilities of archaeal histone involvement in transcription regulation.

## Introduction

Architectural chromatin proteins are found in every domain of life. Bacteria express DNA-bending and DNA-bridging proteins, such as histone-like protein from *Escherichia coli* strain U93 (HU) and histone-like nucleoid-structuring protein (H-NS), to structure and functionally organize the genome and to regulate genome activity [1, 2]. In eukaryotes and most archaeal lineages, histones are responsible for packaging and compaction of the DNA (Table 1). Genomic comparisons demonstrate that the Bacteria and Archaea share a common ancestor; eukaryotes are to date classified as being part of the archaeal branch [3–5]. The archaeal domain comprises single-cellular organisms found in diverse habitats. Although Archaea and Bacteria have common features, such as a circular genome and the absence of a nucleus, at the genetic level, Archaea seem to be more related to eukaryotes. Amongst others, archaeal RNA polymerase, a key component of cellular life in all domains, is more similar to RNA polymerase from eukaryotes than bacterial RNA polymerase [6, 7]. Archaeal ribosomes share their size and structural core with bacterial ribosomes but are more similar to eukaryotic ribosomes when it comes to protein and rRNA sequence and some specific domains [8–10]. Also, some cellular processes thought to be unique to eukaryotes, such as endosomal sorting and the ubiquitin system, have been identified in some archaea [11]. These observations raise the intriguing possibility that chromatin organization as we have come to understand in eukaryotes has evolved from that of the archaeal lineage. Before we describe our analysis, we briefly review current knowledge on chromatin organization in eukaryotes and Archaea and the current paradigms in the evolution of histones, the main chromatin organizing proteins.

**Table 1:** Phylogenetic subdivision of the archaeal domain.

**Superphylum**	**Phylum**	**Class**	**Histones**
**Asgard Archaea**	Candidatus Heimdallarchaeota		Y
	Candidatus Lokiarchaeota		Y
	Candidatus Odinarchaeota		Y
	Candidatus Thorarchaeota		Y
			
**DPANN**	Candidatus Aenigmarchaeota		Y
	Candidatus Diapherotrites		Y
	Candidatus Huberarchaea		Y
	Candidatus Micrarchaeota		Y
	Nanoarchaeota		Y
	Candidatus Nanohaloarchaeota		Y
	Candidatus Pacearchaeota		Y
	Candidatus Parvarchaeota		N
	Candidatus Woesearchaeota		Y
			
**TACK**	Candidatus Bathyarchaeota		Y
	Crenarchaeota		Y*
	Candidatus Geothermarchaeota		N
	Candidatus Korarchaeota		Y
	Thaumarchaeota		Y
	Candidatus Verstraetearchaeota		N
			
**-**	**Euryarchaeota**	Archaeoglobi	Y
		Hadesarchaea	Y
		Halobacteria	Y
		Methanobacteria	Y
		Methanococci	Y
		Methanomicrobia	Y
		Methanonatronarchaeia	Y
		Methanopyri	Y
		Theionarchaea	Y
		Thermococci	Y
		Thermoplasmata	Y

Division of Archaea in superphyla and phyla, including the euryarchaeal classes. Presence (Y) or absence (N) of histone-coding genes on the genome of the members of the phyla and classes have been indicated. An asterisk indicates that histone-coding genes have been found in a minority of species belonging to the phylum.

**Abbreviations:** DPANN, Diapherotrites, Pacearchaeota, Aenigmarchaeota, Nanoarchaeota, Nanohaloarchaeota; TACK, Thaumarchaeota, Aigarchaeota, Crenarchaeota, Korarchaeota.

### The eukaryotic histone

**Fig 1:**
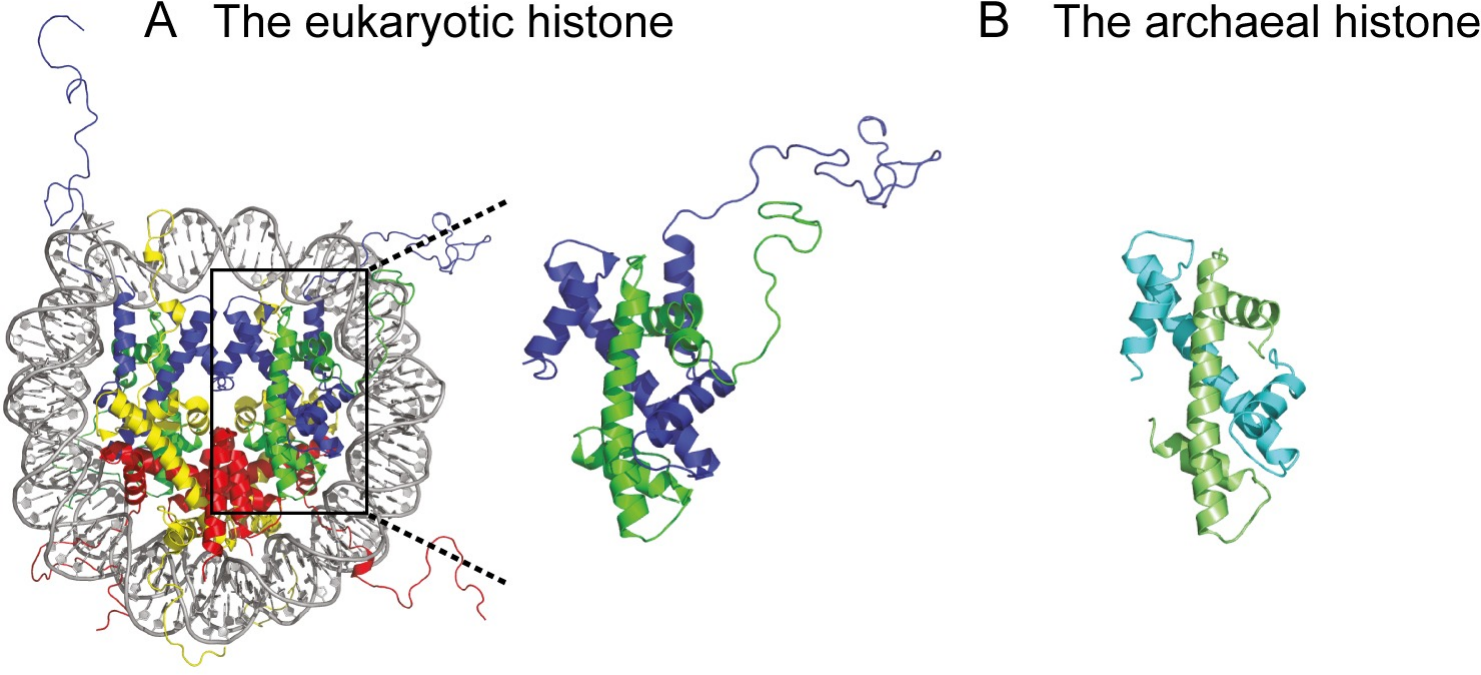
Eukaryotic and archaeal histones. (A) Eukaryotic nucleosome consisting of DNA wrapped around a core of a (H3-H4)_2_ tetramer and two H2A-H2B dimers. Yellow, H2A; red, H2B; blue, H3; green, H4. (B) Archaeal histone homodimer of HMfB. HMfB, Histone B from *Methanothermus fervidus*.

In eukaryotes, octameric histone cores compact DNA by wrapping an approximately 150-bp unit twice around its surface, forming a nucleosome [12, 13]. Nucleosomes interact with each other, yielding an additional level of DNA organization in the form of a fibre. Besides a role in compaction, histones also play roles in genome organization, replication, repair, and expression, which highlights the nucleosome as a very important complex affecting a vast array of cellular processes. Characteristic of core histone proteins of all different origins is a common “histone fold”: two short and one long α-helix, separated by loops [14–18]. In eukaryotes, the histone core consists of two H2A-H2B dimers and a H3-H4 tetramer, around which approximately 146 bp of DNA is wrapped twice (Fig 1A). It has been suggested that smaller histone assemblies, such as tetrasomes (H3-H4 tetramers), hexasomes (H3-H4 tetramers plus one H2A/H2B dimer), and hemisomes (a H3-H4 dimer plus one H2A/H2B dimer), have functional roles as intermediate structures during, for example, transcription elongation [19–22]. The linker histone H1 (which lacks the characteristic histone fold) binds at the entry and exit points of the DNA wrapped around the octameric histone core [23, 24]. The association of histone H1 constrains an additional 20 bp of DNA and allows for the formation of the 30-nm fibre, which results in tighter compaction [25, 26]. Also, flexible N-terminal tails that protrude from eukaryotic histones contribute to tighter DNA packaging. These tails may interact with either the DNA or the histone surface on another nucleosome, which stabilizes the close association of nucleosomes [27–29]. Furthermore, post-translational modifications of amino acid residues in the N-terminal tails, such as acetylation, methylation, phosphorylation, ubiquitination, and biotinylation, are a key instrument for the cell to regulate gene expression, the DNA damage response, and many other processes [30–32]. For instance, while heterochromatin (tightly packed DNA) is typically devoid of acetylated lysines, euchromatic (lightly packed) regions typically contain histones with acetylated lysines. In general, euchromatin contains actively transcribed genes. Histone acetylation is believed to cause a locally less condensed chromatin structure in vivo, which is permissive to transcription. In particular the lysine-rich histone H4 tail seems to be crucial in the modulation of chromatin structure [27]. In vitro, H4 tails are required for higher order chromatin folding [33–35], which can be disrupted by acetylation of K16 [27]. Nucleosome function and level of genome compaction can be altered in a multitude of ways, providing flexible and versatile mechanisms for tuning the cell’s dynamic chromatin structure and transcription regulation.

### Architectural DNA-binding proteins in Archaea

Archaeal genomes also encode proteins that are involved in shaping DNA architecture. Genes coding for histones are found in many species throughout the domain (Table 1). In some species a homologue of the bacterial DNA bender HU was identified [36, 37]. Nucleoid-associated proteins (NAPs) from the Alba family (also known as the *Sulfolobus solfataricus* 10b (Sso10b) protein family) are abundant and widely conserved in Archaea. Notably, Alba family proteins have also been identified in eukaryotes [38]. Characteristic of these proteins is the formation of protein–DNA filaments and bridges between DNA duplexes [39–42]. Two Alba family proteins with different functionalities have been studied in Archaea. Alba1 cooperatively forms filaments in a sequence-independent and concentration-dependent manner in Crenarchaeota, whereas Alba2 only occurs as heterodimer with Alba1 and does not form filaments [38, 42]. Alba proteins have been shown to repress transcription in vitro [43]. In Euryarchaeota, some species express sequence-specific Alba proteins [44], which, like Alba1 homodimers at low-protein concentrations and Alba1-Alba2 heterodimers, may form loops by bridging two DNA duplexes [45]. Other proteins affecting DNA conformation are Sso10a family proteins, which are able to bend and bridge DNA as well as form filaments on DNA [46, 47] and the monomeric DNA benders Cren7 and Sul7 [48, 49]. Cren7 and Sul7 have exclusively been identified in members of the Crenarchaeota phylum, whereas Sso10a has been found in some Crenarchaeota and Euryarchaeota. Other less widespread NAPs include transcription regulator of the maltose system-like 2 (TrmBL2), methanogen chromosomal protein 1 (MC1), *Methanopyrus kandleri* 7 kDa protein (7kMk), *Sulfolobus solfataricus* protein 7c (Sso7c), and crenarchaeal chromatin protein 1 (CC1) [50–55].

**Fig 2:**
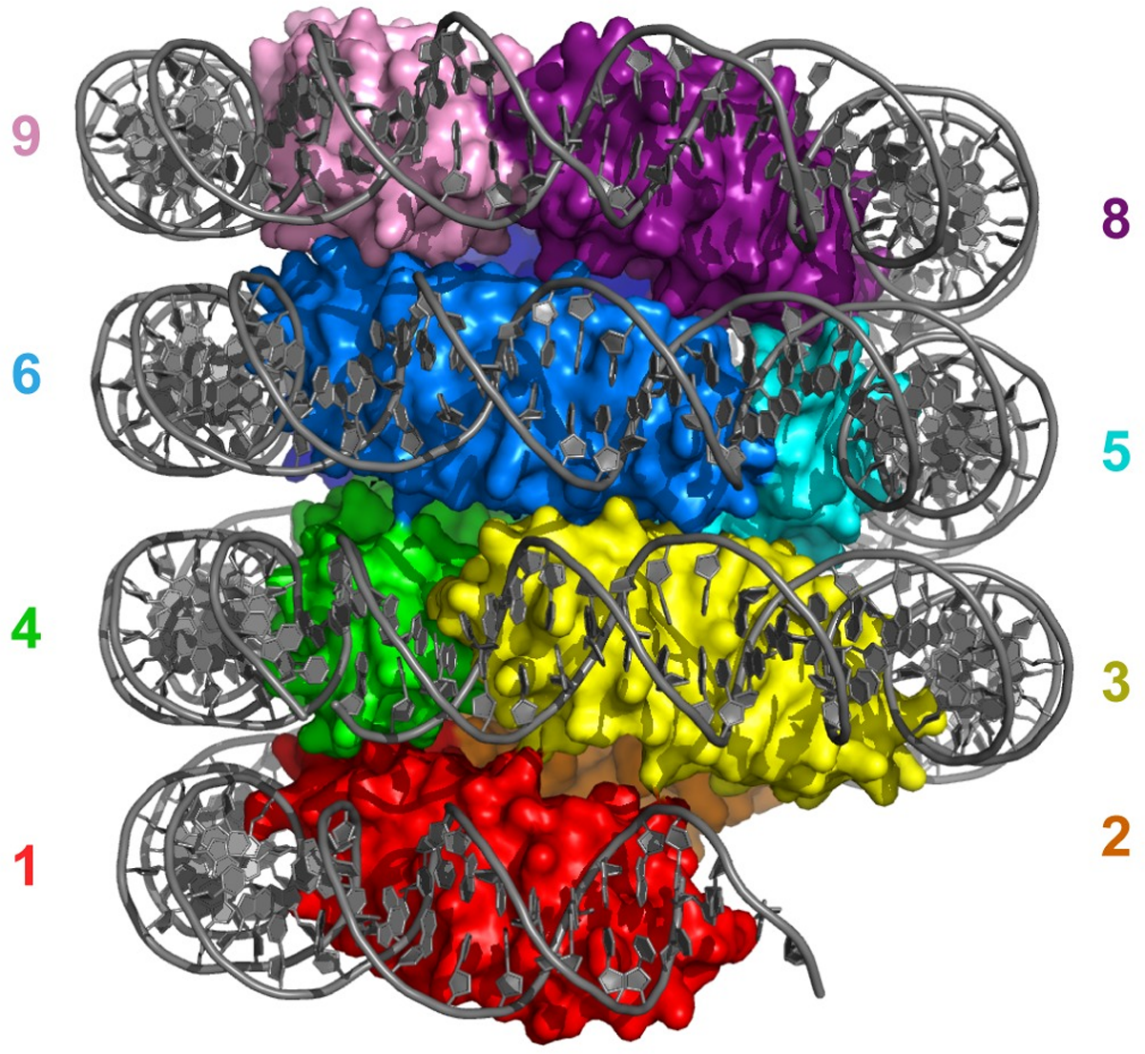
Overview of the hypernucleosome structure. HMfB dimers stack to form a continuous, central protein core that wraps the DNA in a left-handed superhelix. Nine HMfB dimers are shown, each dimer in surface mode and in rainbow colors. Numbering indicates position of the nine histone dimers; note that dimer 5 and 6 occlude the view of dimer 7. DNA is in gray and shown as cartoon. *Image generated using PDB entry 5T5K* [64]. HMfB, Histone B from *Methanothermus fervidus*; PDB, Protein Data Bank.

The histones found in Archaea are widespread throughout the domain but are absent in most Crenarchaeota. They have the same histone fold as eukaryotic histones, but N-terminal histone tails have not been identified (Fig 1B). Linker histones, homologous to eukaryotic H1, have not been found. Archaeal histones exist as dimers in solution, which have been shown to bend DNA [56, 57]. These histone dimers can be homodimeric or heterodimeric [58], as many archaeal species express, or at least encode, more than one histone variant. In *Methanothermus fervidus* (class Methanobacteria), the two histone variants are expressed at different levels and ratios at different growth phases, suggesting a distinct function for both proteins [59]. In addition to binding as dimers, archaeal histones have been reported in vivo and in vitro to bind DNA as tetramers [60–62], wrapping the DNA once. However, micrococcal nuclease (MNase) digestion patterns of *Thermococcus kodakarensis* (class Thermococci) chromatin suggest that histone–DNA complexes consist of discrete multiples of a dimeric histone subunit (i.e., not limited to dimers and tetramers) in vivo without obvious dependence on the DNA sequence [63]. Based on the latter observations, it was proposed that histone dimers multimerize and wrap DNA into a filament of variable length [17, 63]. The crystallography study of Luger and coworkers on histone HMfB from *M*. *fervidus* indicates that these histones assemble into an endless left-handed rod in vitro, which we propose to call a “hypernucleosome” (Fig 2). Note that these complexes were assembled on SELEX-optimized DNA previously shown to favor tetrameric nucleosome assembly [64]. The number of wraps in the hypernucleosome, which is the DNA bending 360° around the histone multimer, scales linearly with the number of histone subunits, resulting in a tight packaging of DNA. The authors also provide evidence that mutation-directed perturbation of hypernucleosome function in vivo alters response to nutrient change in *T*. *kodakarensis*, suggesting a role in transcription. Both eukaryotes and Archaea encode histone proteins, which seem to be involved in response to environmental cues by their involvement in transcription regulation.

### Evolution of the histone protein class

It has been suggested that eukaryotic histones evolved from archaeal histones [65]. This hypothesis is supported by the high similarity at the amino acid sequence level and in secondary structure [66, 67]. Suggestive of an archaeal origin of eukaryotic histones is also the dimeric nature of archaeal histones; archaeal histone complexes are built from dimers, but members of the archaeal class Halobacteria express a “tandem histone.” In these tandem histones, the histone folds are linked end-to-end [68–70]. This implies that the histone folds always occupy the same position and role in the naturally linked dimer. This leads to the relaxation of evolutionary constraints in parts of the histone, an example of subfunctionalization [71, 72]. According to this hypothesis, the histone folds further evolved in a divergent way, leading to an asymmetric dimer. This may have been an ancestor of H3-H4, which later separated to become two individual proteins and corresponding genes [66]. The eukaryotic H3-H4 tetramer resembles the tetramer found in Archaea, and it has been suggested that H2A and H2B have arisen from H3 and H4 later on in histone evolution [66]. Indeed, H3 and H4 are more similar to archaeal histones than H2A and H2B, supporting this hypothesis. From this point, eukaryotic histones have further evolved into histone variants, highly homologous substitutes of canonical eukaryotic histones, which often play a specialist role in a wide variety of cellular processes [73]. Unlike canonical histones, which are mainly expressed during DNA replication, histone variants are expressed in a replication-independent manner [74, 75]. Histone variants of H2A and H3 are widely known and studied, whereas only a few examples have been found of diversified H2B and H4 [76]. The evolutionary pressure for the evolution of dimer-based histones to octameric histones and their subsequent variants was long believed to be DNA compaction [66]. The fact that eukaryotic cells undergo mitosis, in which chromosomes are highly compacted, together with the abundance of gene-poor regions may have favored a histone conformation that wraps DNA twice (eukaryotic octamer) instead of once (archaeal tetramer) and that via its N-terminal tails has the ability to compact DNA at a higher order. Open questions that remain are how histone evolution was driven and what the roles of archaeal histones and their variants are in genome packaging and regulation.

**Fig 3:**
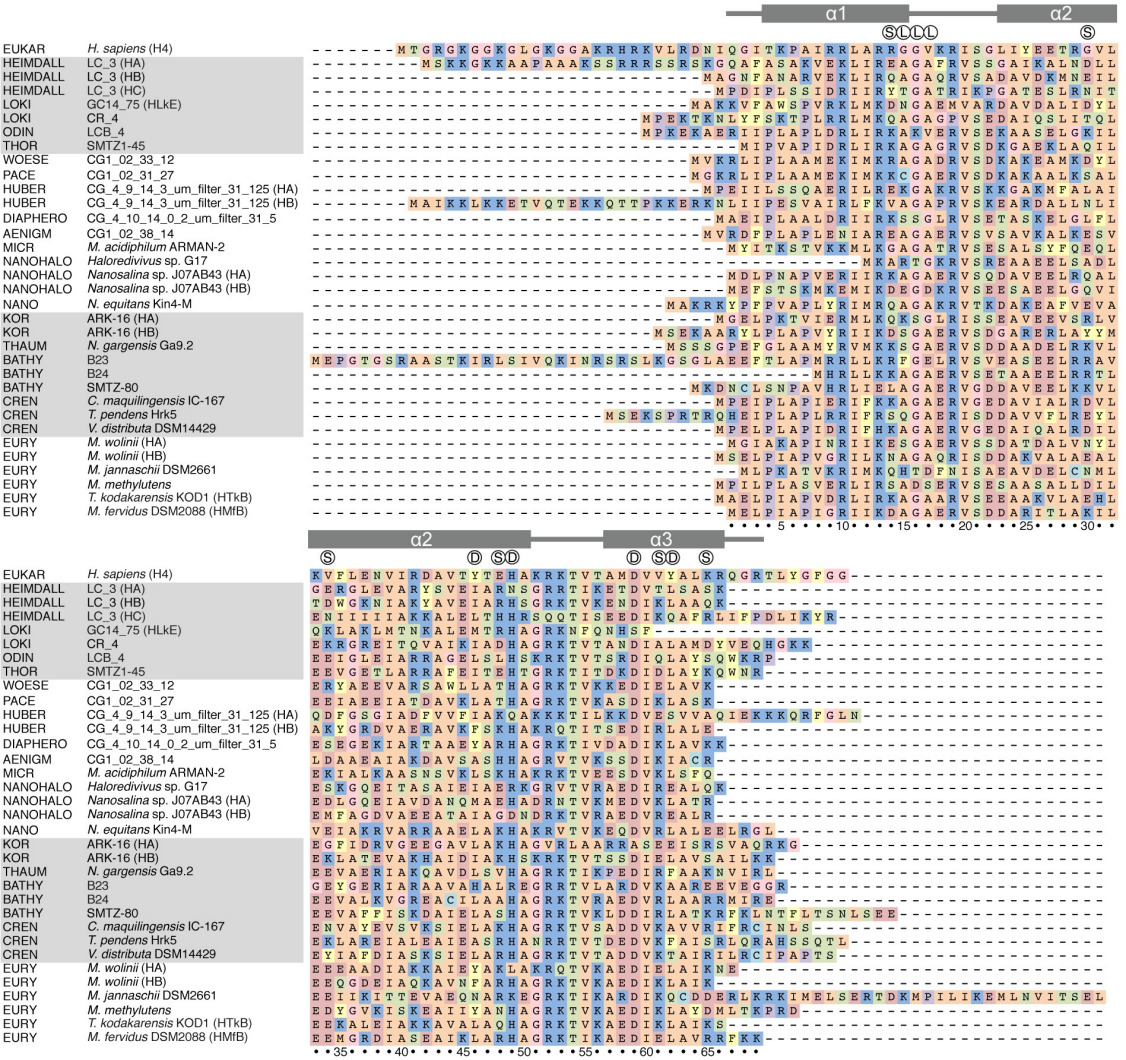
Alignment of histones from different archaeal species and human histone H4. Colors indicate the side chain group: R, H, K: blue; D, E: red; A, V, I, L, M: orange; F, Y, W: yellow; S, T, N, Q: green; C: turquoise; G: pink; P: purple. Symbols above the alignment indicate the dimer–dimer interface (D), the loop of the stacking interface (L), and the putative stacking interactions (S) based on HMfB). Secondary structure and numbering of HMfB is used for reference. EUKAR H4, eukaryotic (human) histone H4 NP_724344.1; HEIMDALL LC_3 HA, HB, and HC, Candidatus Heimdallarchaeota OLS22332.1, OLS24873.1, and OLS21974.1, respectively; LOKI GC14_75 HLkE and CR_4, Candidatus Lokiarchaeota KKK41979.1 and OLS16336.1, respectively; ODIN, Candidatus Odinarchaeota OLS18261.1; THOR, Candidatus Thorarchaeota KXH71038.1; WOESE, Candidatus Woesearchaeota OIO61677.1; PACE, Candidatus Pacearchaeota OIO41945.1; HUBER, Candidatus Huberarchaea CG_4_9_14_3_um_filter_31_125 HA and HB, PJB03565.1, and PJB04497.1, respectively; DIAPHERO, Candidatus Diapherotrites PJA17623.1; AENIGM, Candidatus Aenigmarchaeota OIN88081.1; MICR, Candidatus Micrarchaeota *Micrarchaeum acidiphilum* ARMAN-2 EET90461.1; NANOHALO, Candidatus Nanohaloarchaeota *Haloredivivus* sp. G17 and *Nanosalina* sp. J07AB43 HA and HB, EHK01841.1, EGQ42849.1, and EGQ43804.1, respectively; NANO, Nanoarchaeota *Nanoarchaeum equitans* Kin4-M AAR39197.1; THAUM, Thaumarchaeota *Nitrososphaera gargensis* Ga9.2 AFU59009.1; BATHY B23, B24, and SMTZ-80, Candidatus Bathyarchaeota KYH36356.1, KYH37304.1, and KON27866.1, respectively; CREN, Crenarchaeota *Caldivirga maquilingensis* IC-167, *Thermofilum pendens* Hrk5, and *Vulcanisaeta distribute* DSM14429, ABW02527.1, ABL77757.1, and ADN51226.1, respectively; EURY, Euryarchaeota *Methanobrevibacter wolinii* HA and HB, *Methanocaldococcus jannaschii* DSM2661, *Methanococcoides methylutens*, *Thermococcus kodakarensis* KOD1, and *Methanothermus fervidus* DSM2066, WP_42707783.1, WP_42706862.1, AAB99668.1, KGK98166.1, BAD86478.1, and ADP77985.1, respectively.

Here, we discuss the amino acid residues that are responsible for the formation of the hypernucleosome based on a sequence analysis of a subset of archaeal histones that includes histones from all phyla that contain genes coding for histones (Fig 3). Also, we analyze the ability of histones to form a hypernucleosome and the effects of N- or C-termini longer or shorter than the consensus on histone multimerization and transcription regulation. We emphasize the histones in species from recently discovered phyla, which are believed to be an evolutionary link to eukaryotes [11, 77]. Based on elements that archaeal histones have in common and elements that differ from that consensus, we discuss some of the open questions regarding gene regulation by archaeal histones.

## Histones are found in some newly discovered Archaea

With the widespread use of metagenomic sequencing, entire new branches within the archaeal domain have been discovered. Next to Euryarchaeota, the phylum that has been known since the establishment of Archaea as one of the domains of life [78], the superphyla Thaumarchaeota, Aigarchaeota, Crenarchaeota, Korarchaeota (TACK), Diapherotrites, Pacearchaeota, Aenigmarchaeota, Nanoarchaeota, Nanohaloarchaeota (DPANN), and Asgard Archaea are part of the most recent representation of the tree of life [79]. Genomes of the recently discovered archaeal superphylum Asgard Archaea and candidate phyla Bathyarchaeota, Woesearchaeota, Pacearchaeota, Aenigmarchaeota, Diapherotrites, Huberarchaea, and Micrarchaeota encode histones [11, 77, 80–82](Table 1). With the publication of the genome sequences of these organisms, we were able to scrutinize the sequence divergence of histones by comparing sequences of histones from Archaea throughout the domain (Fig 3). The selection of histones shown here is based on the presence of histone-coding genes in different phyla. Since many of those phyla were only discovered in the last three years, our selection includes a relatively large number of histones that have not yet been studied in vivo or in vitro.

We found that in genome LC_3 of the candidate phylum Heimdallarchaeota, 10 different histones are encoded, which is the highest number of histones found in one archaeal genome [83]. We have not found any histones in the genomes of candidate phyla Parvarchaeota, Geothermarchaeota, and Verstraetearchaeota, although it should be noted that abundance of available genomes and completeness of the genomes differs. The majority of available genomes from the phyla that do not seem to encode histones have an estimated completeness of between 70% and 99% [84–87]. This means that we cannot rule out the possibility that any of those genomes does contain one or more genes coding for histones. The absence of histones suggests that other NAPs may be involved in genome compaction. In that light, it is notable that in genomes from Candidatus Parvarchaeota, as well as in genomes from the candidate phyla from Asgard Archaea, Woesearchaeota, Bathyarchaeota, Pacearchaeota, Aenigmarchaeota, and Micrarchaeota, genes coding for the DNA-bridging protein Alba1 (and in some cases, Alba2) are present. Like histones, Alba (or Sso10b) proteins are likely involved in transcription repression. They are highly abundant in the nonhistone-coding Crenarchaeota [88], possibly taking the functional role of histones as found in other Archaea. Some Parvarchaeota genomes encode Alba but not histones, and their genomes may therefore be shaped or regulated in a similar way as in Crenarchaeota. For Geothermarchaeota and Verstraetearchaeota, we were not able to identify any protein that clearly resembles known chromatin proteins. Furthermore, we found that only Candidatus Thorarchaeota contains an HU gene (a DNA-bending protein generally found in Bacteria and Archaea without histones only [67]). The genome of Candidatus Huberarchaea encodes an MC1 homologue, which is a monomeric DNA-bending protein often found in organisms from the euryarchaeal class Halobacteria. Genes coding for other known archaeal NAPs [50, 89, 90] were not found.

### Some archaeal histones have eukaryote-like N-terminal tails

A striking finding based on the amino acid sequence comparison reveals that two histones from Candidatus Heimdallarchaeota archaeon LC_3 (only Histone A [HA] shown in Fig 3), one from Candidatus Huberarchaea archaeon CG_4_9_14_3_um_filter_31_125 and one from Candidatus Bathyarchaeota archaeon B23, contain an N-terminal tail, which was previously thought to exist only in eukaryotic histones and only recently reported for Heimdallarchaeota [64]. In eukaryotes, these tails stabilize a higher order of compaction by interacting with either the DNA or another nucleosome. The tails of the two histones from Heimdallarchaeota and Huberarchaea are of roughly the same length and sequence composition as eukaryotic H4 tails (see Fig 3). Prompted by the importance of the eukaryotic histone tails in modulating chromatin structure and function [27, 32], we constructed a molecular model of a hypernucleosome formed by Histone A (HA) from Heimdallarchaeota LC_3 to investigate its potential function (see Methods section).

**Fig 4:**
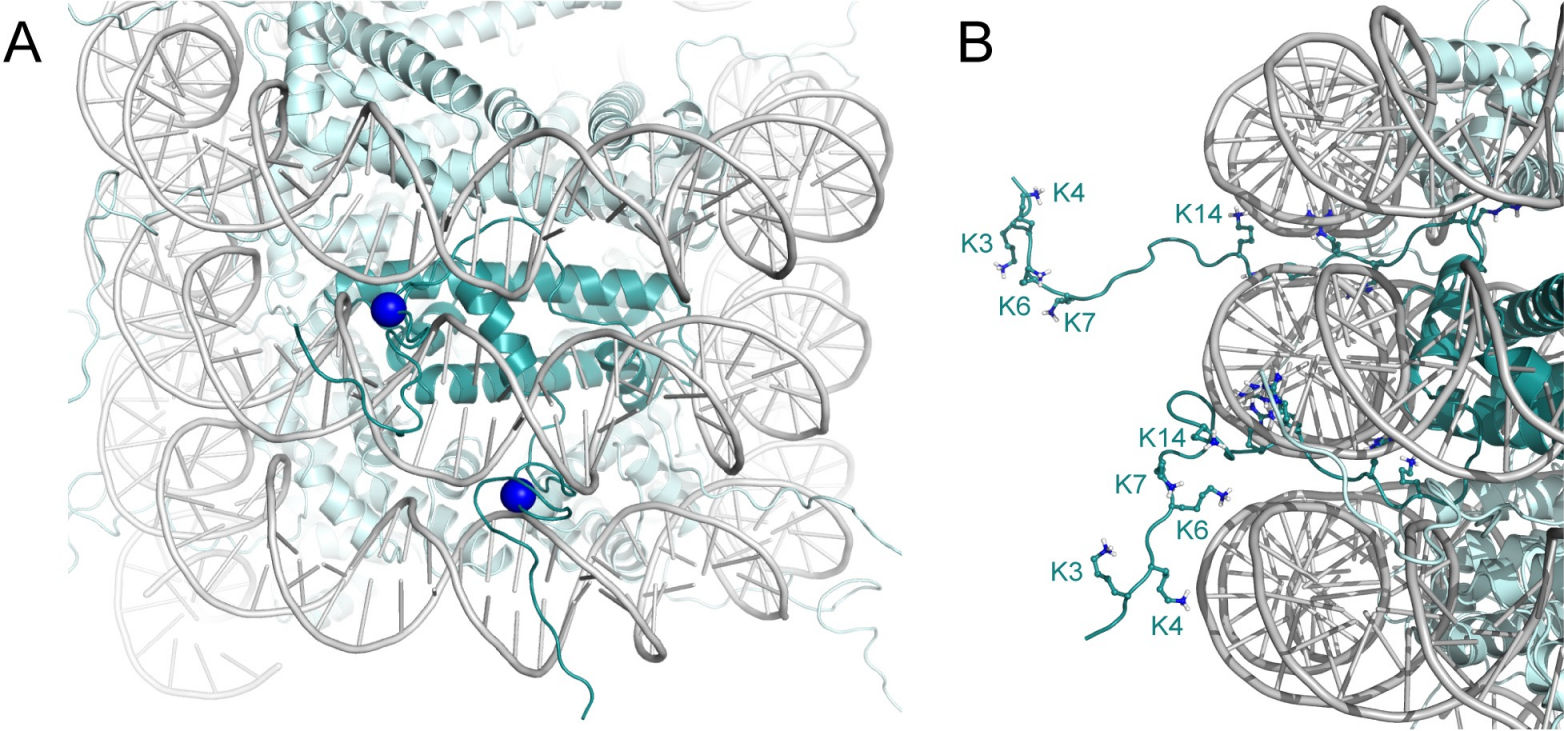
Model of a Heimdall LC_3 hypernucleosome with N-terminal tails. (A) View showing histone tails protruding through the DNA minor grooves. The R17 Cα-atom is shown as a blue sphere to mark the exit point of the tail. (B) Close up of the histone tails with lysine and arginine residues shown as sticks, and N-terminal lysines are labeled. Homodimers of Heimdall LC_3 histone HA are shown in teal; one dimer is highlighted in darker colors. Models are based on the structure of HMfB (PDB entry 5T5K); the tail in the top (bottom) of panel B is modeled in the H3 (H4) tail conformation (PDB entry 1KX5). HA, Histone A; HMfB, Histone B from *Methanothermus fervidus*; PDB, Protein Data Bank.

The model illustrates how three subsequent arginines (R17–R19) could facilitate passing of the tails through the DNA gyres (Fig 4). The tails exit the hypernucleosome through DNA minor grooves, similar to eukaryotic histone tails, and might position their lysine side chains to bind to the hypernucleosomal DNA or to other DNA close by, facilitating (long-range) genomic interactions *in trans*. Like the H4 tail that is subject to acetylation of lysines K5, K8, K12, and K16 [91], lysines in the Heimdallarchaeal histone tail may well be subject to acetylation. Archaeal genomes are known to have several candidate lysine acetyltransferase and deacetylase enzymes, including proteins belonging to the ELP3 superfamily, to which transcription elongation factor and histone acetyltransferase ELP3 belongs [92–94]. Searches using the ProSite database (http://prosite.expasy.org, [95]) and Protein Information Resource (http://pir.georgetown.edu, [96]) further reveal that the Heimdallarchaeota LC_3 genome contains multiple gene products containing the Gcn5-related N-acetyltransferase domain, which is present in many histone acetyltransferases [97]. Interestingly, a potential “reader” protein that binds modified lysines can also be identified. This protein, HeimC3_47440, contains a YEATS-domain, which has recently been shown to bind histone tails that carry acetylated or crotonylated lysines [98–101]. Comparison with the closest homolog of known 3D structure, YEATS2 (35% identity, PDB-id 5IQL, [102]), shows that the binding site for the modified lysine side chain is strictly conserved in the archaeal protein. Notably, only Candidatus Bathyarchaeota, which also features tailed histones, contains a detectable homolog of HeimC3_47440. The presence of lysine-containing N-terminal tails in combination with histone modification writers and readers suggests that Archaea use post-translational modifications in a similar way to Eukaryotes as modulators of genome compaction and gene activity. The tail of the Huberarchaea histone also contains lysine residues that are found at the same position as some of the lysines of the H4 tail. However, no proteins involved in post-translational modification of histone tails have been identified in this phylum.

Other histones, for example from Candidatus Lokiarchaeota CR_4, Candidatus Odinarchaeota LBC_4, *Nanoarchaeum equitans*, and *Thermofilum pendens*, contain a short N-terminal tail of 5–10 residues. Also, histones with a C-terminal tail have been found. The histone from the euryarchaeal species *Methanocaldococcus jannaschii* (class Methanococci) has a 28-residue tail, which seems to be unique among archaeal histones. Other C-terminal tails are up to 11 residues long (as compared to *Methanothermus fervidus* HMfB) and appear in *Caldiarchaeum subterraneum*, Candidatus Bathyarchaeota SMTZ-80, Candidatus Heimdallarchaeota LC_3, Candidatus Lokiarchaeota CR_4, and all histones found in Crenarchaeota. These short C-terminal tails are similar in length to the H4 C-terminal tail, that is reported to play a role in the promotion of histone octamer formation in eukaryotes [103]. The genomes of some archaeal species contain genes for histone truncates. The histone from *Haloredivivus* sp. G17, member of the candidate phylum Nanohaloarchaeota, and the histone from Candidatus Bathyarchaeota archaeon B24 both lack part of the N-terminal α-helix (α1), and one histone from Candidatus Lokiarchaeota GC14-75 is reduced in length at the C-terminus. The remainder of the C-terminal amino acids likely does not form a C-terminal helix (α3) in this histone from Candidatus Lokiarchaeota. Although histones of reduced length or containing tails lack part of the histone fold, they likely still possess DNA-binding properties. Therefore, they possibly have functional roles in the regulation of genes.

### Multimerization of histones

Both eukaryotic histones and HMfB form dimers, a process that is driven by a hydrophobic core (involving residues A24, L28, L32, I39, and A43 in HMfB) as well as a crucial salt bridge for a stable histone fold (R52-D59 in HMfB) [14]. These hydrophobic residues and the salt bridge are conserved among Archaea. This indicates that archaeal histones have very similar tertiary structures [14, 104]. Also, residues that play an important role in DNA binding are present in all examined histones, including the arginines that anchor archaeal histone dimers to the DNA minor grooves (R10 and R19 in HMfB) [14]. Both eukaryotic H3-H4-dimers and HMfB dimers can form tetramers by hydrogen bonding of H49 and D59 (HMfB) and additional hydrophobic interactions in the interface (L46 and L62 in HMfB) [105], pairs of residues that, too, are generally conserved among archaeal histones (Fig 3).

**Fig 5:**
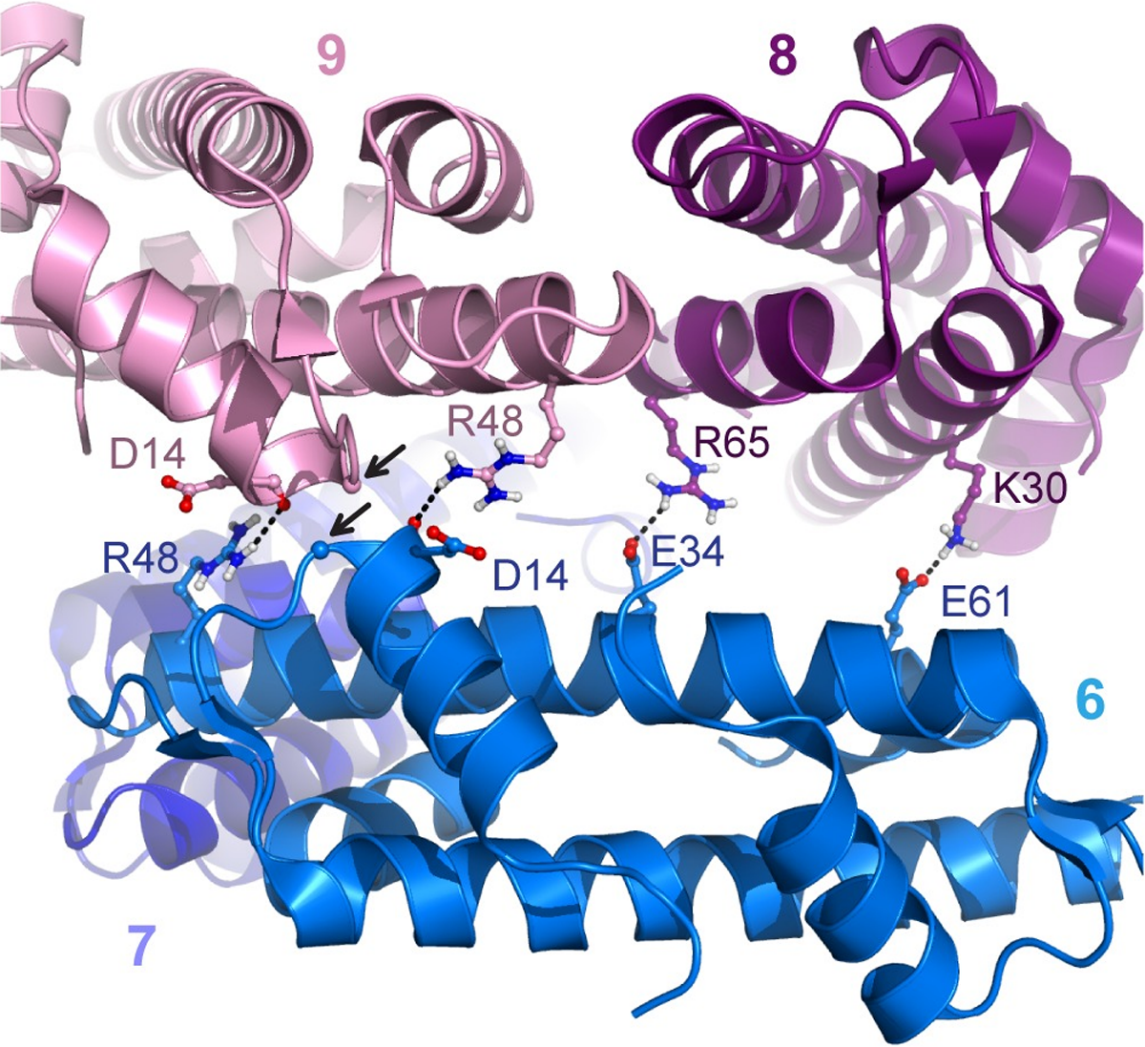
Stacking interface between HMfB dimers in the hypernucleosome. Each dimer *i* forms stacking interactions with dimer *i*+2 and *i*+3, shown here for dimer 6. Residues deemed important are shown in ball-and-sticks and labeled; close stacking of G16 in dimer 6 and 9 is indicated by arrows (Cα shown as spheres); hydrogen-bonds are indicated with dashes. *Image generated using PDB entry 5T5K* [64]. HMfB; Histone B from *Methanothermus fervidus*; PDB, Protein Data Bank.

The HMfB–DNA cocrystal structure reveals left-handed wrapping of DNA around a histone-multimer core [64] (Fig 2). This structure supports the model in which HMfB dimers multimerize along DNA into an “infinite” hypernucleosome, thereby linearly compacting the DNA approximately ten-fold. It is likely that hypernucleosomes grow or shrink by association or dissociation of dimers at both ends. The resolution of the crystal structure allowed us to identify several interacting residues between layers of dimers that may be important for stabilizing the complex (Fig 5). Based on this structural information, the propensity of different archaeal histones to multimerize can be predicted.

In Table 2, we set out three criteria for hypernucleosome formation by archaeal histones. Firstly, conservation of residues in the dimer–dimer interface (L46, H49, D59, and L62 in HMfB) is required, as forming a tetramer is the first step in multimerization. Secondly, residue G16, which is positioned at the stacking interface of the hypernucleosome (Fig 5), is crucial in permitting formation of the hypernucleosome [64]. Bulkier residues at this position interfere with multimerization [64]. Lastly, favorable interactions between histone dimers *i* and *i*+2 and *i*+3, here termed stacking interactions, will contribute to stability of the compacted hypernucleosome. The HMfB hypernucleosome crystal structure shows three stacking interactions, hydrogen bonds from K30 to E61, E34 to R65, and R48 to D14 (Figs 3 and 5).

**Table 2:** Assessment of possible hypernucleosome formation by archaeal histones.

		Dimer–dimer interface^a^	Stacking interface^b^	Potential stacking interactions^c^	Hypernucleosome formation	Histone features
Heimdall	LC_3 HA	±	+	3 (E14-R48, K26-E57, R41-E45)	**±**	N-terminal tail
Heimdall	LC_3 HB	+	+	5 (E30-K61, Q14-R48, R13-Q18, K27-E57, K37-E45)	**+**	
Heimdall	LC_3 HC	±	+	3 (N34-R65, T15-K41, Y14-Q53)	**±**	
Loki	GC14_75 (HLkE)	−	+	2 (D14-R48, K34-E45)	**−**	Truncated C-term
Loki	CR_4	+	+	2 (Q14-D48, Q41-Q41)	**+**	
Odin	LCB_4	+	−	3 (K30-Q61, K14-E18, E38-R41)	**±**	
Thor	SMTZ1-45	+	+	5 (Q30-D61, E34-K65, K14-E48, E37-R41, E26-K58)	**+**	
Woese	CG1_02_33_12	+	+	4 (R14-T48, R34-E61, E26-K57, E37-R41)	**+**	
Pace	CG1_02_31_27	+	+	4 (S30-K61, E34-K65, K14-T48, E37-K45)	**+**	
Huber	CG_4_9_14_3_um_filter_31_125 (HA)	±	+	2 (E14-K48, E14-K18)	**±**	
Huber	CG_4_9_14_3_um_filter_31_125 (HB)	+	+	2 (N30-R61, K34-E65)	**+**	N-terminal tail
Diaphero	CG_4_10_14_0_2_um_filter_31_5	±	±	4 (E33-R48, E37-R41, E37-R48, E27-K61)	**±**	
Aenigm	CG1_02_38_14	+	+	5 (E30-K61, D34-R65, E14-H48, A15-K41, E37-K41)	**+**	
Micr	*M*. *acidiphilum* ARMAN-2	+	+	3 (E30-K61, K34-Q65, Y2-K48)	**+**	
Nanohalo	*Haloredivivus* sp. G17	±	−	2 (E27-R61, Q37-E45)	**−**	Truncated N-term
Nanohalo	*Nanosalina* sp. J07AB43 (HA)	+	+	5 (Q30-K61, D34-R65, K14-E48, K14-E18, Q37-Q45)	**+**	
Nanohalo	*Nanosalina* sp. J07AB43 (HB)	−	±	2 (Q30-R61, D14-K18)	**−**	
Nano	*N*. *equitans* Kin4-M	+	+	4 (E30-R61, Q14-K48, Q14(bb)-R41, K37-E45)	**+**	
Kor	ARK-16 (HA)	±	±	7 (R30-E61, Q14-K48, K15-E41, E26-R58, E27-R57, E33-K48, R38-E41)	**±**	
Kor	ARK-16 (HB)	+	+	3 (Y30-E61, D14-K48, R27-E61)	**+**	
Thaum	*N*. *gargensis* Ga9.2	+	+	4 (E34-K65, K14-E18, E27-R61, E37-K41)	**+**	
Bathy	B23	±	±	4 (R14-V44, E34-K61, E37-R41, E26-R58)	**±**	N-terminal tail
Bathy	B24	+	+	3 (E34-R65, K14-E18, E27-R61)	**+**	Truncated N-term
Bathy	SMTZ-80	+	+	3 (E34-K65, K41-E45, E27-R61)	**+**	
Cren	*C*. *maquilingensis* IC-167	+	+	4 (D30-K61, N34-R65, K14-E18, Y37-K48)	**+**	
Cren	*T*. *pendens* Hrk5	+	+	4 (E30-K61, S14-R48, R37-E45, R13-E18)	**+**	
Cren	*V*. *distributa* DSM14429	+	+	4 (D30-K61, Y34-R65, K14(bb)-R48, K14-E18)	**+**	
Eury	*M*. *wolinii* (HA)	±	+	4 (N30-E61, E34-K65, E14-K48, K41-E45)	**±**	
Eury	*M*. *wolinii* (HB)	+	+	5 (E30-K61, E34-K65, N14-R48, N14-Q18, Q41-Q41)	**+**	
Eury	*M*. *jannaschii* DSM2661	+	−	4 (N30-K61, Q14-R48, K37-Q45, D26-R58)	**±**	C-terminal tail
Eury	*M*. *methylutens*	±	−	2 (D30-K61, S14-E18)	**−**	
Eury	*T*. *kodakarensis* KOD1 (HTkB)	+	+	4 (E30-K61, E34-K65, K14-Q48, K26-E58)	**+**	
Eury	*M*. *fervidus* DSM2088 (HMfB)	+	+	3 (K30-E61, E34-R65, D14-R48)	**+**	

Dimer–dimer interactions in the tetrameric interface are expected to be essential for hypernucleosome formation. Absence of bulky residues in the first loop and a high number of potential hydrogen bonds in the stacking interface will enhance the compactness and stability of the hypernucleosome. Likely, uncertain and unlikely stacking ability is indicated with +, ±, and −, respectively.

^a^ Dimer–dimer interface includes residues at positions 46, 49, 59, and 62.

^b^ Stacking interface includes residues at positions 15–17.

^c^ For all potential stacking interactions, residue numbering of HMfB was used according to the alignment in Fig 3.

**Abbreviations:** HA, Histone A; HB, Histone B; HLkE, Histone E from Lokiarchaeota; HMfB, Histone B from *Methanothermus fervidus*; HTkB, Histone B from *Thermococcus kodakarensis*.

Scrutiny of histone sequences reveals that most archaeal histones meet these criteria and are thus likely to form hypernucleosomes (Table 2, marked +). We identified two to seven potential stacking interactions for this group of histones, which may affect hypernucleosome stability and compactness. Fewer interactions may allow for more “breathing” of the hypernucleosome structure, yielding hypernucleosomes that are more flexible or “floppy.” We predict such structures to be formed also by a number of archaeal histones that do not fully meet our criteria (Table 2, marked ±). For example, Candidatus Heimdallarchaeota LC_3 HA and Candidatus Lokiarchaeota GC14_75 HLkE have H49N and D59S substitutions, respectively, which likely weakens the crucial hydrogen-bonding interaction at the dimer–dimer interface [105]. Similarly, substitution of the hydrophobic residues 46 and 62 for more hydrophilic or bulkier ones would lead to a less stable dimer–dimer interface, as for Candidatus Heimdallarchaeota LC_3 HC and Candidatus Bathyarchaeota B23. In the presence of the canonical dimer–dimer interface, bulky substitutions at position 16 likely also result in a more open hypernucleosome structure, as for Candidatus Odinarchaeota LCB_4.

Three archaeal histone species fail multiple criteria in our analysis, indicating that these cannot form hypernucleosomes. These histone species are *Haloredivivus* sp G17, *Nanosalina* J07AB43 HB, and Euryarchaeal *Methanococcoides methylutens* (class Methanomicrobia) that all combine defects in the dimer interface with a bulky substitution at position 16 and few potential stacking interactions (Table 2, marked–). In particular, *Nanosalina* J07AB43 Histone B (HB) shows a H49D substitution and a glutamic acid at position 62, making the dimer surface highly negatively charged and thus very unlikely to interact with another dimer.

It is remarkable that most of the histones having N- or C-terminal tails or N- or C-terminal truncations additionally have substitutions in the dimer–dimer and/or stacking interface that will affect hypernucleosome formation. Histones with reduced ability to form compact hypernucleosomes are expected to exhibit different roles in shaping the genome, like simple DNA bending or site-specific interference with histone multimerization. Interestingly, the genomes of several organisms encode histones that we predict are able to multimerize as well as histones that probably do not multimerize. This suggests that they may, in addition to directly binding to promoters, also be able to affect gene regulation by multimerization.

### Histones in genome regulation

MNase-seq experiments have shown that histones position upstream and downstream of a promoter region [106]. This, in combination with knock-out studies showing both up- and down-regulation of transcription levels, leads to the hypothesis that histones are important for transcription regulation in the relatively well-studied phylum Euryarchaeota [45, 69, 107, 108] and may play a similar role in other histone-coding phyla. The exact mechanisms by which histones act in regulation are at this moment largely unknown. What is the mechanistic role of histones in the regulation of gene expression? Is the hypernucleosome, with a mechanism analogous to that in bacterial gene repression, able to block promoter regions and other regulatory elements, thereby making them inaccessible to the transcription machinery [109–112]? In Bacteria, such a mechanism exists for H-NS and partition protein B (ParB) proteins, in which filaments laterally spread from a nucleation site, often a high-affinity DNA sequence [113–116]. Specific high-affinity sites have been identified both in vivo and in vitro in Archaea [61, 106, 117, 118]. The role of such high-affinity sites may be to position the hypernucleosome on the genome and could be a key feature in archaeal genome regulation. In Archaea, cooperative lateral spreading of filaments has been reported for Alba proteins [40, 42, 119, 120]. Also, promoter occlusion mechanisms and competitive binding of archaeal NAPs and transcription factors have been reported [45, 121, 122].

In addition, how dynamic are hypernucleosomes, and how does the cell control the size of the hypernucleosome in order for it to be functional? Is up- and down-regulation of histone expression important in fine tuning this process? Another option for control of hypernucleosome size is heteromerization of histone variants with different stacking propensity. Heteromerization of such histone variants, for instance HA and HB from *Nanosalina* J07AB43 (Table 2), could restrict hypernucleosome size to fewer subunits. Distinct expression patterns of histone variants at different growth phases or as a result of environmental cues such as osmolarity [59, 107], may alter the composition and size of the hypernucleosome. However, so far, histone variants have been poorly studied in Archaea. The results of our predictions on hypernucleosome formation clearly point out the need for in vitro and in vivo studies explicitly addressing all of these questions.

## Conclusion

Histones from Archaea and eukaryotes are similar in tertiary but not in quaternary structure when bound to DNA. While eukaryotic histones form octamers on the DNA, archaeal histones form filaments of variable size: hypernucleosomes. Important residues responsible for DNA binding, dimer–dimer interactions, and stacking interactions are mostly conserved among Archaea, including Asgard Archaea, Bathyarchaeota, and other newly discovered Archaea. In these recently discovered Archaeal phyla, histone tails and truncated histone variants were also found. In terms of evolution, it appears that, based on fragmentary data derived from extant lineages, the hypernucleosome has progressively become more flexible as histones with N-terminal and C-terminal tails and additional terminal helices (like in H2A and H2B in the nucleosome) developed. Furthermore, the appearance of additional DNA-binding residues and positively charged N-terminal tails may have increased the affinity of histones for DNA [123]. These changes in dimer structure and DNA affinity may have stabilized octameric nucleosomes and disfavored multimerization. Specifically, the emergence of the eukaryotic H2A-H2B heterodimer blocked hypernucleosome formation since H2A lacks the dimer–dimer interface, and H2B contains an additional helix at its C-terminus that blocks the stacking interface.

The histone tails from Candidatus Heimdallarchaeota are likely to function in similar ways as those of eukaryotic histones. They are lysine rich and potentially subject to post-translational modification, thereby possibly affecting the histone’s interactions with other actors. Alternatively, they may provide stabilization of the hypernucleosome via interactions with DNA *in cis* or *in trans*. Since it is believed that eukaryotes share their latest common ancestor with Candidatus Heimdallarchaeota, eukaryotic histones may have evolved from the predecessors of the tail-containing Heimdallarchaeal histones. As some histone proteins that have an N-terminal tail (Candidatus Heimdallarchaeota LC_3 HA and Bathyarchaeota archaeon B23) seem to form less stable hypernucleosomes, these histones may represent an evolutionary transition towards a different mechanism of gene regulation, switching from regulation by multimerization and compaction toward regulation by histone tail modifications.

Although the hypernucleosome structure is suggestive of stacking interactions between dimers in adjacent turns, experimental evidence for such interactions is lacking. Also, the functional role of tails, as well as truncates, has yet to be proven experimentally. In vitro hypernucleosome reconstitution experiments and in vivo foot-printing assays of species expressing nonstandard histones combined with mutation of the residues proposed to be involved in stacking interactions could answer these questions. Lastly, the existence of post-translational modifications of residues in archaeal histone tails, as well as their effect on transcription regulation, remains to be discovered and would give an important insight into the evolution of transcription regulation and genome folding from Archaea to eukaryotes.

## Methods

### Selection and alignment of archaeal histone sequences

We have included histones from every histone-encoding (candidate) phylum within the archaeal domain in our analysis. We show different histones from the same organism if the predicted stacking properties are very dissimilar. Sequences were aligned with Clustal Omega [124] using default parameters, removing gaps.

### Analysis of potential hypernucleosome formation

Structural analysis of the selected archaeal histones and assessment of potential hypernucleosome formation was done by inspecting the conservation of residues that are important for multimerization in the published HMfB hypernucleosome structure [64]. Comparative multichain modeling was performed in MODELLER [125] using default parameters to construct dimer models of the archaeal histones. These models were superimposed onto HMfB dimers in the hypernucleosome crystal structure to assess whether alternative or additional interactions were possible in the different archaeal histone complexes.

### Model of Heimdall HA tails in hypernucleosome

The molecular model of the histone HA dimer from the Heimdallarchaeota LC_3 genome was constructed by multitemplate modeling in MODELLER [125] using otherwise default parameters. The HMfB dimer in the hypernucleosome [64] was used as a structural template for the histone fold and eukaryotic histone H3 and H4 as structural templates for the N-terminal tails. An initial model for the Heimdall HA hypernucleosome was obtained by superimposing the HA dimer model onto HMfB in the hypernucleosome crystal structure, with either an H3-like or an H4-like tail conformation. To optimize the path of the tails through the DNA gyres and remove major steric clashes, the HA dimer model and surrounding DNA was excised from the initial model and water refined separately using High-Ambiguity Driven Docking (HADDOCK) [126], imposing ambiguous interaction restraints between HA residues 14–19 and the surrounding 3-bp section of DNA, using otherwise default parameters.

## References

[1] Dorman CJ. Genome architecture and global gene regulation in bacteria: making progress towards a unified model? Nat Rev Microbiol. 2013;11(5):349–55. 10.1038/nrmicro3007 . 23549066

[2] Dame RT, Tark-Dame M. Bacterial chromatin: converging views at different scales. Curr Opin Cell Biol. 2016;40:60–5. 10.1016/j.ceb.2016.02.015 . 26942688

[3] Eme L, Spang A, Lombard J, Stairs CW, Ettema TJG. Archaea and the origin of eukaryotes. Nat Rev Microbiol. 2017;15(12):711–23. 10.1038/nrmicro.2017.133 . 29123225

[4] Williams TA, Foster PG, Cox CJ, Embley TM. An archaeal origin of eukaryotes supports only two primary domains of life. Nature. 2013;504(7479):231–6. 10.1038/nature12779 . 24336283

[5] Cox CJ, Foster PG, Hirt RP, Harris SR, Embley TM. The archaebacterial origin of eukaryotes. Proc Natl Acad Sci U S A. 2008;105(51):20356–61. 10.1073/pnas.0810647105 ; PubMed Central PMCID: PMCPMC2629343. 19073919 PMC2629343

[6] Huet J, Schnabel R, Sentenac A, Zillig W. Archaebacteria and eukaryotes possess DNA-dependent RNA polymerases of a common type. EMBO J. 1983;2(8):1291–4. ; PubMed Central PMCID: PMCPMC555274. 10872322 10.1002/j.1460-2075.1983.tb01583.x PMC555274

[7] Lane WJ, Darst SA. Molecular evolution of multisubunit RNA polymerases: structural analysis. J Mol Biol. 2010;395(4):686–704. 10.1016/j.jmb.2009.10.063 ; PubMed Central PMCID: PMCPMC2813324. 19895816 PMC2813324

[8] Armache JP, Anger AM, Marquez V, Franckenberg S, Frohlich T, Villa E, et al Promiscuous behaviour of archaeal ribosomal proteins: implications for eukaryotic ribosome evolution. Nucleic Acids Res. 2013;41(2):1284–93. 10.1093/nar/gks1259 ; PubMed Central PMCID: PMCPMC3553981. 23222135 PMC3553981

[9] Petrov AS, Bernier CR, Hsiao C, Norris AM, Kovacs NA, Waterbury CC, et al Evolution of the ribosome at atomic resolution. Proc Natl Acad Sci U S A. 2014;111(28):10251–6. 10.1073/pnas.1407205111 ; PubMed Central PMCID: PMCPMC4104869. 24982194 PMC4104869

[10] Yutin N, Puigbo P, Koonin EV, Wolf YI. Phylogenomics of prokaryotic ribosomal proteins. PLoS ONE. 2012;7(5):e36972 10.1371/journal.pone.0036972 ; PubMed Central PMCID: PMCPMC3353972. 22615861 PMC3353972

[11] Zaremba-Niedzwiedzka K, Caceres EF, Saw JH, Backstrom D, Juzokaite L, Vancaester E, et al Asgard archaea illuminate the origin of eukaryotic cellular complexity. Nature. 2017;541(7637):353–8. 10.1038/nature21031 . 28077874

[12] Luger K, Mader AW, Richmond RK, Sargent DF, Richmond TJ. Crystal structure of the nucleosome core particle at 2.8 A resolution. Nature. 1997;389(6648):251–60. 10.1038/38444 . 9305837

[13] Kornberg RD. Chromatin structure: a repeating unit of histones and DNA. Science. 1974;184(4139):868–71. . 4825889 10.1126/science.184.4139.868

[14] Decanniere K, Babu AM, Sandman K, Reeve JN, Heinemann U. Crystal structures of recombinant histones HMfA and HMfB from the hyperthermophilic archaeon Methanothermus fervidus. J Mol Biol. 2000;303(1):35–47. 10.1006/jmbi.2000.4104 . 11021968

[15] Sandman K, Reeve JN. Archaeal histones and the origin of the histone fold. Curr Opin Microbiol. 2006;9(5):520–5. 10.1016/j.mib.2006.08.003 . 16920388

[16] Arents G, Burlingame RW, Wang BC, Love WE, Moudrianakis EN. The nucleosomal core histone octamer at 3.1 A resolution: a tripartite protein assembly and a left-handed superhelix. Proc Natl Acad Sci U S A. 1991;88(22):10148–52. ; PubMed Central PMCID: PMCPMC52885. 1946434 10.1073/pnas.88.22.10148 PMC52885

[17] Henneman B, Dame RT. Archaeal histones: dynamic and versatile genome architects. AIMS Microbiol. 2015;1(1):72–81. 10.3934/microbiol.2015.1.72 PubMed PMID: WOS:000215290400005.

[18] Malik HS, Henikoff S. Phylogenomics of the nucleosome. Nat Struct Biol. 2003;10(11):882–91. 10.1038/nsb996 PubMed PMID: WOS:000186229100006. 14583738

[19] Katan AJ, Vlijm R, Lusser A, Dekker C. Dynamics of nucleosomal structures measured by high-speed atomic force microscopy. Small. 2015;11(8):976–84. 10.1002/smll.201401318 . 25336288

[20] Levchenko V, Jackson B, Jackson V. Histone release during transcription: displacement of the two H2A-H2B dimers in the nucleosome is dependent on different levels of transcription-induced positive stress. Biochemistry. 2005;44(14):5357–72. 10.1021/bi047786o . 15807529

[21] Hamiche A, Carot V, Alilat M, De Lucia F, O'Donohue MF, Revet B, et al Interaction of the histone (H3-H4)2 tetramer of the nucleosome with positively supercoiled DNA minicircles: Potential flipping of the protein from a left- to a right-handed superhelical form. Proc Natl Acad Sci U S A. 1996;93(15):7588–93. ; PubMed Central PMCID: PMCPMC38790. 8755519 10.1073/pnas.93.15.7588 PMC38790

[22] Arimura Y, Tachiwana H, Oda T, Sato M, Kurumizaka H. Structural analysis of the hexasome, lacking one histone H2A/H2B dimer from the conventional nucleosome. Biochemistry. 2012;51(15):3302–9. 10.1021/bi300129b 22448809

[23] Bednar J, Garcia-Saez I, Boopathi R, Cutter AR, Papai G, Reymer A, et al Structure and Dynamics of a 197 bp Nucleosome in Complex with Linker Histone H1. Mol Cell. 2017;66(3):384–97 e8. 10.1016/j.molcel.2017.04.012 ; PubMed Central PMCID: PMCPMC5508712. 28475873 PMC5508712

[24] Zhou BR, Feng HQ, Kato H, Dai L, Yang YD, Zhou YQ, et al Structural insights into the histone H1-nucleosome complex. P Natl Acad Sci USA. 2013;110(48):19390–5. 10.1073/pnas.1314905110 PubMed PMID: WOS:000327390400064. 24218562 PMC3845106

[25] Cutter AR, Hayes JJ. Linker histones: novel insights into structure-specific recognition of the nucleosome. Biochem Cell Biol. 2017;95(2):171–8. 10.1139/bcb-2016-0097 ; PubMed Central PMCID: PMCPMC5654525. 28177778 PMC5654525

[26] Robinson PJ, Rhodes D. Structure of the '30 nm' chromatin fibre: a key role for the linker histone. Curr Opin Struct Biol. 2006;16(3):336–43. 10.1016/j.sbi.2006.05.007 . 16714106

[27] Shogren-Knaak M, Ishii H, Sun JM, Pazin MJ, Davie JR, Peterson CL. Histone H4-K16 acetylation controls chromatin structure and protein interactions. Science. 2006;311(5762):844–7. 10.1126/science.1124000 . 16469925

[28] Pepenella S, Murphy KJ, Hayes JJ. A distinct switch in interactions of the histone H4 tail domain upon salt-dependent folding of nucleosome arrays. J Biol Chem. 2014;289(39):27342–51. 10.1074/jbc.M114.595140 ; PubMed Central PMCID: PMCPMC4175364. 25122771 PMC4175364

[29] Zhang R, Erler J, Langowski J. Histone Acetylation Regulates Chromatin Accessibility: Role of H4K16 in Inter-nucleosome Interaction. Biophys J. 2017;112(3):450–9. 10.1016/j.bpj.2016.11.015 ; PubMed Central PMCID: PMCPMC5300776. 27931745 PMC5300776

[30] Zhao Y, Garcia BA. Comprehensive Catalog of Currently Documented Histone Modifications. Cold Spring Harb Perspect Biol. 2015;7(9):a025064 10.1101/cshperspect.a025064 ; PubMed Central PMCID: PMCPMC4563710. 26330523 PMC4563710

[31] Jenuwein T, Allis CD. Translating the histone code. Science. 2001;293(5532):1074–80. 10.1126/science.1063127 . 11498575

[32] Kouzarides T. Chromatin modifications and their function. Cell. 2007;128(4):693–705. 10.1016/j.cell.2007.02.005 . 17320507

[33] Kalashnikova AA, Porter-Goff ME, Muthurajan UM, Luger K, Hansen JC. The role of the nucleosome acidic patch in modulating higher order chromatin structure. J R Soc Interface. 2013;10(82):20121022 10.1098/rsif.2012.1022 ; PubMed Central PMCID: PMCPMC3627075. 23446052 PMC3627075

[34] Zhou J, Fan JY, Rangasamy D, Tremethick DJ. The nucleosome surface regulates chromatin compaction and couples it with transcriptional repression. Nat Struct Mol Biol. 2007;14(11):1070–6. 10.1038/nsmb1323 . 17965724

[35] Dorigo B, Schalch T, Bystricky K, Richmond TJ. Chromatin fiber folding: requirement for the histone H4 N-terminal tail. J Mol Biol. 2003;327(1):85–96. . 12614610 10.1016/s0022-2836(03)00025-1

[36] Stein DB, Searcy DG. Physiologically important stabilization of DNA by a prokaryotic histone-like protein. Science. 1978;202(4364):219–21. . 694528 10.1126/science.694528

[37] Searcy DG, Delange RJ. Thermoplasma acidophilum histone-like protein. Partial amino acid sequence suggestive of homology to eukaryotic histones. Biochim Biophys Acta. 1980;609(1):197–200. . 7407184 10.1016/0005-2787(80)90212-9

[38] Goyal M, Banerjee C, Nag S, Bandyopadhyay U. The Alba protein family: Structure and function. Biochim Biophys Acta. 2016;1864(5):570–83. 10.1016/j.bbapap.2016.02.015 . 26900088

[39] Forterre P, Confalonieri F, Knapp S. Identification of the gene encoding archeal-specific DNA-binding proteins of the Sac10b family. Mol Microbiol. 1999;32(3):669–70. . 10320587 10.1046/j.1365-2958.1999.01366.x

[40] Jelinska C, Petrovic-Stojanovska B, Ingledew WJ, White MF. Dimer-dimer stacking interactions are important for nucleic acid binding by the archaeal chromatin protein Alba. Biochem J. 2010;427(1):49–55. 10.1042/BJ20091841 ; PubMed Central PMCID: PMCPMC2841500. 20082605 PMC2841500

[41] Lurz R, Grote M, Dijk J, Reinhardt R, Dobrinski B. Electron microscopic study of DNA complexes with proteins from the Archaebacterium Sulfolobus acidocaldarius. EMBO J. 1986;5(13):3715–21. ; PubMed Central PMCID: PMCPMC1167416. 16453745 10.1002/j.1460-2075.1986.tb04705.x PMC1167416

[42] Laurens N, Driessen RP, Heller I, Vorselen D, Noom MC, Hol FJ, et al Alba shapes the archaeal genome using a delicate balance of bridging and stiffening the DNA. Nat Commun. 2012;3:1328 10.1038/ncomms2330 ; PubMed Central PMCID: PMCPMC3535426. 23271660 PMC3535426

[43] Bell SD, Botting CH, Wardleworth BN, Jackson SP, White MF. The interaction of Alba, a conserved archaeal chromatin protein, with Sir2 and its regulation by acetylation. Science. 2002;296(5565):148–51. 10.1126/science.1070506 . 11935028

[44] Liu Y, Guo L, Guo R, Wong RL, Hernandez H, Hu J, et al The Sac10b homolog in Methanococcus maripaludis binds DNA at specific sites. J Bacteriol. 2009;191(7):2315–29. 10.1128/JB.01534-08 ; PubMed Central PMCID: PMCPMC2655493. 19168623 PMC2655493

[45] Peeters E, Driessen RP, Werner F, Dame RT. The interplay between nucleoid organization and transcription in archaeal genomes. Nat Rev Microbiol. 2015;13(6):333–41. 10.1038/nrmicro3467 . 25944489

[46] Driessen RP, Lin SN, Waterreus WJ, van der Meulen AL, van der Valk RA, Laurens N, et al Diverse architectural properties of Sso10a proteins: Evidence for a role in chromatin compaction and organization. Sci Rep. 2016;6:29422 10.1038/srep29422 ; PubMed Central PMCID: PMCPMC4941522. 27403582 PMC4941522

[47] Kahsai MA, Vogler B, Clark AT, Edmondson SP, Shriver JW. Solution structure, stability, and flexibility of Sso10a: a hyperthermophile coiled-coil DNA-binding protein. Biochemistry. 2005;44(8):2822–32. 10.1021/bi047669t 15723526

[48] Edmondson SP, Shriver JW. DNA binding proteins Sac7d and Sso7d from Sulfolobus. Methods Enzymol. 2001;334:129–45. . 11398456 10.1016/s0076-6879(01)34463-4

[49] Guo L, Feng Y, Zhang Z, Yao H, Luo Y, Wang J, et al Biochemical and structural characterization of Cren7, a novel chromatin protein conserved among Crenarchaea. Nucleic Acids Res. 2008;36(4):1129–37. 10.1093/nar/gkm1128 ; PubMed Central PMCID: PMCPMC2275093. 18096617 PMC2275093

[50] Driessen RP, Dame RT. Nucleoid-associated proteins in Crenarchaea. Biochem Soc Trans. 2011;39(1):116–21. 10.1042/BST0390116 . 21265758

[51] Maruyama H, Shin M, Oda T, Matsumi R, Ohniwa RL, Itoh T, et al Histone and TK0471/TrmBL2 form a novel heterogeneous genome architecture in the hyperthermophilic archaeon Thermococcus kodakarensis. Mol Biol Cell. 2011;22(3):386–98. 10.1091/mbc.E10-08-0668 ; PubMed Central PMCID: PMCPMC3031468. 21148291 PMC3031468

[52] Culard F, Laine B, Sautiere P, Maurizot JC. Stoichiometry of the binding of chromosomal protein MC1 from the archaebacterium, Methanosarcina spp. CHTI55, to DNA. FEBS Lett. 1993;315(3):335–9. . 8422927 10.1016/0014-5793(93)81189-7

[53] Pavlov NA, Cherny DI, Nazimov IV, Slesarev AI, Subramaniam V. Identification, cloning and characterization of a new DNA-binding protein from the hyperthermophilic methanogen Methanopyrus kandleri. Nucleic Acids Res. 2002;30(3):685–94. ; PubMed Central PMCID: PMCPMC100301. 11809880 10.1093/nar/30.3.685 PMC100301

[54] Oppermann UC, Knapp S, Bonetto V, Ladenstein R, Jornvall H. Isolation and structure of repressor-like proteins from the archaeon Sulfolobus solfataricus. Co-purification of RNase A with Sso7c. FEBS Lett. 1998;432(3):141–4. . 9720912 10.1016/s0014-5793(98)00848-5

[55] Luo X, Schwarz-Linek U, Botting CH, Hensel R, Siebers B, White MF. CC1, a novel crenarchaeal DNA binding protein. J Bacteriol. 2007;189(2):403–9. 10.1128/JB.01246-06 ; PubMed Central PMCID: PMCPMC1797387. 17085561 PMC1797387

[56] Bailey KA, Marc F, Sandman K, Reeve JN. Both DNA and histone fold sequences contribute to archaeal nucleosome stability. J Biol Chem. 2002;277(11):9293–301. 10.1074/jbc.M110029200 . 11751933

[57] Sandman K, Krzycki JA, Dobrinski B, Lurz R, Reeve JN. HMf, a DNA-binding protein isolated from the hyperthermophilic archaeon Methanothermus fervidus, is most closely related to histones. Proc Natl Acad Sci U S A. 1990;87(15):5788–91. ; PubMed Central PMCID: PMCPMC54413. 2377617 10.1073/pnas.87.15.5788 PMC54413

[58] Nishida H, Oshima T. Archaeal histone distribution is associated with archaeal genome base composition. J Gen Appl Microbiol. 2017;63(1):28–35. 10.2323/jgam.2016.07.003 . 27990001

[59] Sandman K, Grayling RA, Dobrinski B, Lurz R, Reeve JN. Growth-phase-dependent synthesis of histones in the archaeon Methanothermus fervidus. Proc Natl Acad Sci U S A. 1994;91(26):12624–8. ; PubMed Central PMCID: PMCPMC45491. 7809089 10.1073/pnas.91.26.12624 PMC45491

[60] Pereira SL, Grayling RA, Lurz R, Reeve JN. Archaeal nucleosomes. Proc Natl Acad Sci U S A. 1997;94(23):12633–7. ; PubMed Central PMCID: PMCPMC25063. 9356501 10.1073/pnas.94.23.12633 PMC25063

[61] Bailey KA, Pereira SL, Widom J, Reeve JN. Archaeal histone selection of nucleosome positioning sequences and the procaryotic origin of histone-dependent genome evolution. J Mol Biol. 2000;303(1):25–34. 10.1006/jmbi.2000.4128 . 11021967

[62] van der Valk RA, Laurens N, Dame RT. Tethered Particle Motion Analysis of the DNA Binding Properties of Architectural Proteins. Methods Mol Biol. 2017;1624:127–43. 10.1007/978-1-4939-7098-8_11 . 28842881

[63] Maruyama H, Harwood JC, Moore KM, Paszkiewicz K, Durley SC, Fukushima H, et al An alternative beads-on-a-string chromatin architecture in Thermococcus kodakarensis. EMBO Rep. 2013;14(8):711–7. 10.1038/embor.2013.94 ; PubMed Central PMCID: PMCPMC3736136. 23835508 PMC3736136

[64] Mattiroli F, Bhattacharyya S, Dyer PN, White AE, Sandman K, Burkhart BW, et al Structure of histone-based chromatin in Archaea. Science. 2017;357(6351):609–12. 10.1126/science.aaj1849 . 28798133 PMC5747315

[65] Arents G, Moudrianakis EN. The histone fold: a ubiquitous architectural motif utilized in DNA compaction and protein dimerization. Proc Natl Acad Sci U S A. 1995;92(24):11170–4. ; PubMed Central PMCID: PMCPMC40593. 7479959 10.1073/pnas.92.24.11170 PMC40593

[66] Malik HS, Henikoff S. Phylogenomics of the nucleosome. Nat Struct Biol. 2003;10(11):882–91. 10.1038/nsb996 . 14583738

[67] Sandman K, Pereira SL, Reeve JN. Diversity of prokaryotic chromosomal proteins and the origin of the nucleosome. Cell Mol Life Sci. 1998;54(12):1350–64. 10.1007/s000180050259 . 9893710 PMC11147202

[68] Ng WV, Kennedy SP, Mahairas GG, Berquist B, Pan M, Shukla HD, et al Genome sequence of Halobacterium species NRC-1. Proc Natl Acad Sci U S A. 2000;97(22):12176–81. 10.1073/pnas.190337797 ; PubMed Central PMCID: PMCPMC17314. 11016950 PMC17314

[69] Dulmage KA, Todor H, Schmid AK. Growth-Phase-Specific Modulation of Cell Morphology and Gene Expression by an Archaeal Histone Protein. MBio. 2015;6(5):e00649–15. 10.1128/mBio.00649-15 ; PubMed Central PMCID: PMCPMC4600100. 26350964 PMC4600100

[70] Becker EA, Seitzer PM, Tritt A, Larsen D, Krusor M, Yao AI, et al Phylogenetically driven sequencing of extremely halophilic archaea reveals strategies for static and dynamic osmo-response. PLoS Genet. 2014;10(11):e1004784 10.1371/journal.pgen.1004784 ; PubMed Central PMCID: PMCPMC4230888. 25393412 PMC4230888

[71] Stoltzfus A. On the possibility of constructive neutral evolution. J Mol Evol. 1999;49(2):169–81. . 10441669 10.1007/pl00006540

[72] Lynch M, Force A. The probability of duplicate gene preservation by subfunctionalization. Genetics. 2000;154(1):459–73. ; PubMed Central PMCID: PMCPMC1460895. 10629003 10.1093/genetics/154.1.459 PMC1460895

[73] Ausio J. Histone variants—the structure behind the function. Brief Funct Genomic Proteomic. 2006;5(3):228–43. 10.1093/bfgp/ell020 . 16772274

[74] Marzluff WF, Duronio RJ. Histone mRNA expression: multiple levels of cell cycle regulation and important developmental consequences. Curr Opin Cell Biol. 2002;14(6):692–9. . 12473341 10.1016/s0955-0674(02)00387-3

[75] Weber CM, Henikoff S. Histone variants: dynamic punctuation in transcription. Genes Dev. 2014;28(7):672–82. 10.1101/gad.238873.114 ; PubMed Central PMCID: PMCPMC4015494. 24696452 PMC4015494

[76] Henikoff S, Smith MM. Histone variants and epigenetics. Cold Spring Harb Perspect Biol. 2015;7(1):a019364 10.1101/cshperspect.a019364 ; PubMed Central PMCID: PMCPMC4292162. 25561719 PMC4292162

[77] Spang A, Saw JH, Jorgensen SL, Zaremba-Niedzwiedzka K, Martijn J, Lind AE, et al Complex archaea that bridge the gap between prokaryotes and eukaryotes. Nature. 2015;521(7551):173–9. 10.1038/nature14447 ; PubMed Central PMCID: PMCPMC4444528. 25945739 PMC4444528

[78] Woese CR, Fox GE. Phylogenetic structure of the prokaryotic domain: the primary kingdoms. Proc Natl Acad Sci U S A. 1977;74(11):5088–90. ; PubMed Central PMCID: PMCPMC432104. 270744 10.1073/pnas.74.11.5088 PMC432104

[79] Spang A, Caceres EF, Ettema TJG. Genomic exploration of the diversity, ecology, and evolution of the archaeal domain of life. Science. 2017;357(6351). 10.1126/science.aaf3883 . 28798101

[80] He Y, Li M, Perumal V, Feng X, Fang J, Xie J, et al Genomic and enzymatic evidence for acetogenesis among multiple lineages of the archaeal phylum Bathyarchaeota widespread in marine sediments. Nat Microbiol. 2016;1(6):16035 10.1038/nmicrobiol.2016.35 . 27572832

[81] Probst AJ, Castelle CJ, Singh A, Brown CT, Anantharaman K, Sharon I, et al Genomic resolution of a cold subsurface aquifer community provides metabolic insights for novel microbes adapted to high CO2 concentrations. Environ Microbiol. 2017;19(2):459–74. 10.1111/1462-2920.13362 . 27112493

[82] Probst AJ, Ladd B, Jarett JK, Geller-McGrath DE, Sieber CMK, Emerson JB, et al Differential depth distribution of microbial function and putative symbionts through sediment-hosted aquifers in the deep terrestrial subsurface. Nat Microbiol. 2018;3(3):328–36. 10.1038/s41564-017-0098-y . 29379208 PMC6792436

[83] Fricke WF, Seedorf H, Henne A, Kruer M, Liesegang H, Hedderich R, et al The genome sequence of Methanosphaera stadtmanae reveals why this human intestinal archaeon is restricted to methanol and H2 for methane formation and ATP synthesis. J Bacteriol. 2006;188(2):642–58. 10.1128/JB.188.2.642-658.2006 ; PubMed Central PMCID: PMCPMC1347301. 16385054 PMC1347301

[84] Rinke C, Schwientek P, Sczyrba A, Ivanova NN, Anderson IJ, Cheng JF, et al Insights into the phylogeny and coding potential of microbial dark matter. Nature. 2013;499(7459):431–7. 10.1038/nature12352 . 23851394

[85] Parks DH, Rinke C, Chuvochina M, Chaumeil PA, Woodcroft BJ, Evans PN, et al Recovery of nearly 8,000 metagenome-assembled genomes substantially expands the tree of life. Nat Microbiol. 2017;2(11):1533–42. 10.1038/s41564-017-0012-7 . 28894102

[86] Jungbluth SP, Amend JP, Rappe MS. Metagenome sequencing and 98 microbial genomes from Juan de Fuca Ridge flank subsurface fluids. Sci Data. 2017;4:170037 10.1038/sdata.2017.37 ; PubMed Central PMCID: PMCPMC5369317. 28350381 PMC5369317

[87] Vanwonterghem I, Evans PN, Parks DH, Jensen PD, Woodcroft BJ, Hugenholtz P, et al Methylotrophic methanogenesis discovered in the archaeal phylum Verstraetearchaeota. Nat Microbiol. 2016;1:16170 10.1038/nmicrobiol.2016.170 . 27694807

[88] Xue H, Guo R, Wen YF, Liu DX, Huang L. An abundant DNA binding protein from the hyperthermophilic archaeon Sulfolobus shibatae affects DNA supercoiling in a temperature-dependent fashion. Journal of Bacteriology. 2000;182(14):3929–33. 10.1128/Jb.182.14.3929–3933.2000 PubMed PMID: WOS:000087938500007. 10869069 PMC94576

[89] Driessen RP, Dame RT. Structure and dynamics of the crenarchaeal nucleoid. Biochem Soc Trans. 2013;41(1):321–5. 10.1042/BST20120336 . 23356305

[90] White MF, Bell SD. Holding it together: chromatin in the Archaea. Trends Genet. 2002;18(12):621–6. . 12446147 10.1016/s0168-9525(02)02808-1

[91] O'Neill LP, Turner BM. Histone H4 acetylation distinguishes coding regions of the human genome from heterochromatin in a differentiation-dependent but transcription-independent manner. EMBO J. 1995;14(16):3946–57. ; PubMed Central PMCID: PMCPMC394473. 7664735 10.1002/j.1460-2075.1995.tb00066.x PMC394473

[92] Reeve JN. Archaeal chromatin and transcription. Mol Microbiol. 2003;48(3):587–98. . 12694606 10.1046/j.1365-2958.2003.03439.x

[93] Eichler J, Adams MWW. Posttranslational protein modification in Archaea. Microbiol Mol Biol R. 2005;69(3):393–425. 10.1128/Mmbr.69.3.393–425.2005 PubMed PMID: WOS:000231838800002. PMC1197805 16148304

[94] Beltrao P, Bork P, Krogan NJ, van Noort V. Evolution and functional cross-talk of protein post-translational modifications. Mol Syst Biol. 2013;9. doi: ARTN 714 10.1002/msb.201304521 PubMed PMID: WOS:000342502000002. 24366814 PMC4019982

[95] Sigrist CJA, Cerutti L, de Castro E, Langendijk-Genevaux PS, Bulliard V, Bairoch A, et al PROSITE, a protein domain database for functional characterization and annotation. Nucleic Acids Research. 2010;38:D161–D6. 10.1093/nar/gkp885 PubMed PMID: WOS:000276399100026. 19858104 PMC2808866

[96] Wu CH, Yeh LSL, Huang HZ, Arminski L, Castro-Alvear J, Chen YX, et al The Protein Information Resource. Nucleic Acids Research. 2003;31(1):345–7. 10.1093/nar/gkg040 PubMed PMID: WOS:000181079700083. 12520019 PMC165487

[97] Berger SL. Gene activation by histone and factor acetyltransferases. Curr Opin Cell Biol. 1999;11(3):336–41. 10.1016/S0955-0674(99)80046-5 PubMed PMID: WOS:000080799100007. 10395565

[98] Li YY, Wen H, Xi YX, Tanaka K, Wang HB, Peng DN, et al AF9 YEATS Domain Links Histone Acetylation to DOT1L-Mediated H3K79 Methylation. Cell. 2014;159(3):558–71. 10.1016/j.cell.2014.09.049 PubMed PMID: WOS:000344521700012. 25417107 PMC4344132

[99] Shanle EK, Andrews FH, Meriesh H, McDaniel SL, Dronamraju R, DiFiore JV, et al Association of Taf14 with acetylated histone H3 directs gene transcription and the DNA damage response. Gene Dev. 2015;29(17):1795–800. 10.1101/gad.269977.115 PubMed PMID: WOS:000361415700003. 26341557 PMC4573853

[100] Andrews FH, Shinsky SA, Shanle EK, Bridgers JB, Gest A, Tsun IK, et al The Taf14 YEATS domain is a reader of histone crotonylation. Nat Chem Biol. 2016;12(6):396–U33. 10.1038/nchembio.2065 PubMed PMID: WOS:000376160600007. 27089029 PMC4871749

[101] Li YY, Sabari BR, Panchenko T, Wen H, Zhao D, Guan HP, et al Molecular Coupling of Histone Crotonylation and Active Transcription by AF9 YEATS Domain. Mol Cell. 2016;62(2):181–93. 10.1016/j.molcel.2016.03.028 PubMed PMID: WOS:000374643900005. 27105114 PMC4841940

[102] Zhao D, Guan HP, Zhao S, Mi WY, Wen H, Li YY, et al YEATS2 is a selective histone crotonylation reader. Cell Res. 2016;26(5):629–32. PubMed PMID: WOS:000377449200011. 10.1038/cr.2016.49 27103431 PMC4856769

[103] Chavez MS, Scorgie JK, Dennehey BK, Noone S, Tyler JK, Churchill ME. The conformational flexibility of the C-terminus of histone H4 promotes histone octamer and nucleosome stability and yeast viability. Epigenetics Chromatin. 2012;5(1):5 10.1186/1756-8935-5-5 ; PubMed Central PMCID: PMCPMC3439350. 22541333 PMC3439350

[104] Fahrner RL, Cascio D, Lake JA, Slesarev A. An ancestral nuclear protein assembly: crystal structure of the Methanopyrus kandleri histone. Protein Sci. 2001;10(10):2002–7. 10.1110/ps.10901 ; PubMed Central PMCID: PMCPMC2374223. 11567091 PMC2374223

[105] Marc F, Sandman K, Lurz R, Reeve JN. Archaeal histone tetramerization determines DNA affinity and the direction of DNA supercoiling. J Biol Chem. 2002;277(34):30879–86. 10.1074/jbc.M203674200 . 12058041

[106] Nalabothula N, Xi L, Bhattacharyya S, Widom J, Wang JP, Reeve JN, et al Archaeal nucleosome positioning in vivo and in vitro is directed by primary sequence motifs. BMC Genomics. 2013;14:391 10.1186/1471-2164-14-391 ; PubMed Central PMCID: PMCPMC3691661. 23758892 PMC3691661

[107] Wilkinson SP, Ouhammouch M, Geiduschek EP. Transcriptional activation in the context of repression mediated by archaeal histones. Proc Natl Acad Sci U S A. 2010;107(15):6777–81. 10.1073/pnas.1002360107 ; PubMed Central PMCID: PMCPMC2872413. 20351259 PMC2872413

[108] Xie Y, Reeve JN. Transcription by an archaeal RNA polymerase is slowed but not blocked by an archaeal nucleosome. J Bacteriol. 2004;186(11):3492–8. 10.1128/JB.186.11.3492-3498.2004 ; PubMed Central PMCID: PMCPMC415759. 15150236 PMC415759

[109] Ueguchi C, Kakeda M, Mizuno T. Autoregulatory Expression of the Escherichia-Coli-Hns Gene Encoding a Nucleoid Protein—H-Ns Functions as a Repressor of Its Own Transcription. Mol Gen Genet. 1993;236(2–3):171–8. 10.1007/Bf00277109 PubMed PMID: WOS:A1993KK98500003. 8437561

[110] Dorman CJ. H-NS: A universal regulator for a dynamic genome. Nature Reviews Microbiology. 2004;2(5):391–400. 10.1038/nrmicro883 PubMed PMID: WOS:000221589700013. 15100692

[111] Baek JH, Rajagopala SV, Chattoraj DK. Chromosome segregation proteins of Vibrio cholerae as transcription regulators. MBio. 2014;5(3):e01061–14. 10.1128/mBio.01061-14 ; PubMed Central PMCID: PMCPMC4010829. 24803519 PMC4010829

[112] Ringgaard S, Ebersbach G, Borch J, Gerdes K. Regulatory cross-talk in the double par locus of plasmid pB171. J Biol Chem. 2007;282(5):3134–45. 10.1074/jbc.M609092200 . 17092933

[113] Breier AM, Grossman AD. Whole-genome analysis of the chromosome partitioning and sporulation protein Spo0J (ParB) reveals spreading and origin-distal sites on the Bacillus subtilis chromosome. Mol Microbiol. 2007;64(3):703–18. 10.1111/j.1365-2958.2007.05690.x . 17462018

[114] Pratto F, Cicek A, Weihofen WA, Lurz R, Saenger W, Alonso JC. Streptococcus pyogenes pSM19035 requires dynamic assembly of ATP-bound ParA and ParB on parS DNA during plasmid segregation. Nucleic Acids Res. 2008;36(11):3676–89. 10.1093/nar/gkn170 ; PubMed Central PMCID: PMCPMC2441792. 18477635 PMC2441792

[115] van der Valk RA, Vreede J, Qin L, Moolenaar GF, Hofmann A, Goosen N, et al Mechanism of environmentally driven conformational changes that modulate H-NS DNA-bridging activity. Elife. 2017;6 10.7554/eLife.27369 ; PubMed Central PMCID: PMCPMC5647153. 28949292 PMC5647153

[116] Lang B, Blot N, Bouffartigues E, Buckle M, Geertz M, Gualerzi CO, et al High-affinity DNA binding sites for H-NS provide a molecular basis for selective silencing within proteobacterial genomes. Nucleic Acids Res. 2007;35(18):6330–7. 10.1093/nar/gkm712 ; PubMed Central PMCID: PMCPMC2094087. 17881364 PMC2094087

[117] Ammar R, Torti D, Tsui K, Gebbia M, Durbic T, Bader GD, et al Chromatin is an ancient innovation conserved between Archaea and Eukarya. Elife. 2012;1. doi: ARTN e00078 10.7554/eLife.00078 PubMed PMID: WOS:000328584600004. 23240084 PMC3510453

[118] Tompitak M, Vaillant C, Schiessel H. Genomes of Multicellular Organisms Have Evolved to Attract Nucleosomes to Promoter Regions. Biophys J. 2017;112(3):505–11. 10.1016/j.bpj.2016.12.041 PubMed PMID: WOS:000393734300012. 28131316 PMC5300838

[119] Zhao K, Chai X, Marmorstein R. Structure of a Sir2 substrate, Alba, reveals a mechanism for deacetylation-induced enhancement of DNA binding. J Biol Chem. 2003;278(28):26071–7. 10.1074/jbc.M303666200 . 12730210

[120] Visone V, Vettone A, Serpe M, Valenti A, Perugino G, Rossi M, et al Chromatin structure and dynamics in hot environments: architectural proteins and DNA topoisomerases of thermophilic archaea. Int J Mol Sci. 2014;15(9):17162–87. 10.3390/ijms150917162 ; PubMed Central PMCID: PMCPMC4200833. 25257534 PMC4200833

[121] Grohmann D, Werner F. Recent advances in the understanding of archaeal transcription. Current Opinion in Microbiology. 2011;14(3):328–34. 10.1016/j.mib.2011.04.012 PubMed PMID: WOS:000292948300016. 21596617

[122] Sheppard C, Werner F. Structure and mechanisms of viral transcription factors in archaea. Extremophiles. 2017;21(5):829–38. 10.1007/s00792-017-0951-1 PubMed PMID: WOS:000408230000001. 28681113 PMC5569661

[123] Soares DJ, Marc F, Reeve JN. Conserved eukaryotic histone-fold residues substituted into an archaeal histone increase DNA affinity but reduce complex flexibility. J Bacteriol. 2003;185(11):3453–7. 10.1128/JB.185.11.3453-3457.2003 ; PubMed Central PMCID: PMCPMC155370. 12754245 PMC155370

[124] Sievers F, Wilm A, Dineen D, Gibson TJ, Karplus K, Li W, et al Fast, scalable generation of high-quality protein multiple sequence alignments using Clustal Omega. Mol Syst Biol. 2011;7:539 10.1038/msb.2011.75 ; PubMed Central PMCID: PMCPMC3261699. 21988835 PMC3261699

[125] Webb B, Sali A. Comparative Protein Structure Modeling Using MODELLER. Curr Protoc Bioinformatics. 2014;47:5 6 1–32. 10.1002/0471250953.bi0506s47 . 25199792

[126] Dominguez C, Boelens R, Bonvin AM. HADDOCK: a protein-protein docking approach based on biochemical or biophysical information. J Am Chem Soc. 2003;125(7):1731–7. 10.1021/ja026939x . 12580598

